# *Disporum nanchuanense* (Colchicaceae), a new species from Chongqing, China

**DOI:** 10.3897/phytokeys.130.34005

**Published:** 2019-08-29

**Authors:** Xinxin Zhu, Shuai Liao, Sirong Yi

**Affiliations:** 1 College of Life Sciences, Xinyang Normal University, Xinyang, Henan, 464000, China; 2 Chongqing Three Gorges Medical College, Chongqing Engineering Research Center of Antitumor Natural Drugs, Wanzhou 404120, China; 3 School of Life Sciences, East China Normal University, Shanghai 200241, China; 4 Institute for Conservation and Utilization of Agro-bioresources in Dabie Mountains, Xinyang Normal University, Xinyang, 464000, China

**Keywords:** Jinfo Mountain, Liliaceae, morphology, taxonomy

## Abstract

*Disporum nanchuanense* (Colchicaceae), a new species from Jinfo Mountain National Nature Reserve, Nanchuan District, Chongqing, China, is described and illustrated. It is similar to *D. longistylum* and *D. megalanthum*, but differs from the former in its stem branched type, tepals colour and size, stamens and pistil size; and it differs from the latter in inflorescence position, tepals shape, stamens position, pistil position and size. Meanwhile, the new taxon is assessed as Vulnerable (VU D2), according to the IUCN Red List criteria. Furthermore, an identification key to all Chinese species of *Disporum* is provided.

## Introduction

*Disporum*Salisbury (1812) includes about 24 species distributed in Bhutan, China, India, Japan, Korea, Laos, Malaysia, Myanmar, Nepal, Russia, Sikkim, Thailand and Vietnam (Liang and Tamura 2000; Li et al. 2007; Hu et al. 2016; Zhu et al. 2016; Hareesh et al. 2018; WCSPF 2018). According to Liang and Tamura (2000), there are 14 species in China, including 8 endemic species. Recently, three new species were described by Li et al. (2007), Hu et al. (2016), and Zhu et al. (2016) from China.

During three field expeditions in Jinfo Mountain of Nanchuan District, Chongqing, an unknown species of *Disporum* was collected. After careful studies of the genus, particularly the flower characteristics of those species in the adjacent regions, as well as comparison amongst the unknown species and their related species, we conclude that it is a new species of *Disporum*, which has usually a simple stem, terminal inflorescence, large flower, white tepals with purple, apex obtuse and distinctly exserted stamens and style. A detailed description, along with line drawings, photographs, habitat, distribution and conservation status, as well as morphological comparison to similar species are also provided. Furthermore, a key to Chinese species of *Disporum* is provided.

## Taxonomy

### Disporum nanchuanense

Taxon classificationPlantaeLilialesColchicaceae

X.X.Zhu & S.R.Yi sp. nov.

6188C3141E9D5BAFA1A62A40E90A981B

urn:lsid:ipni.org:names:60479342-2

**Figure 1.**
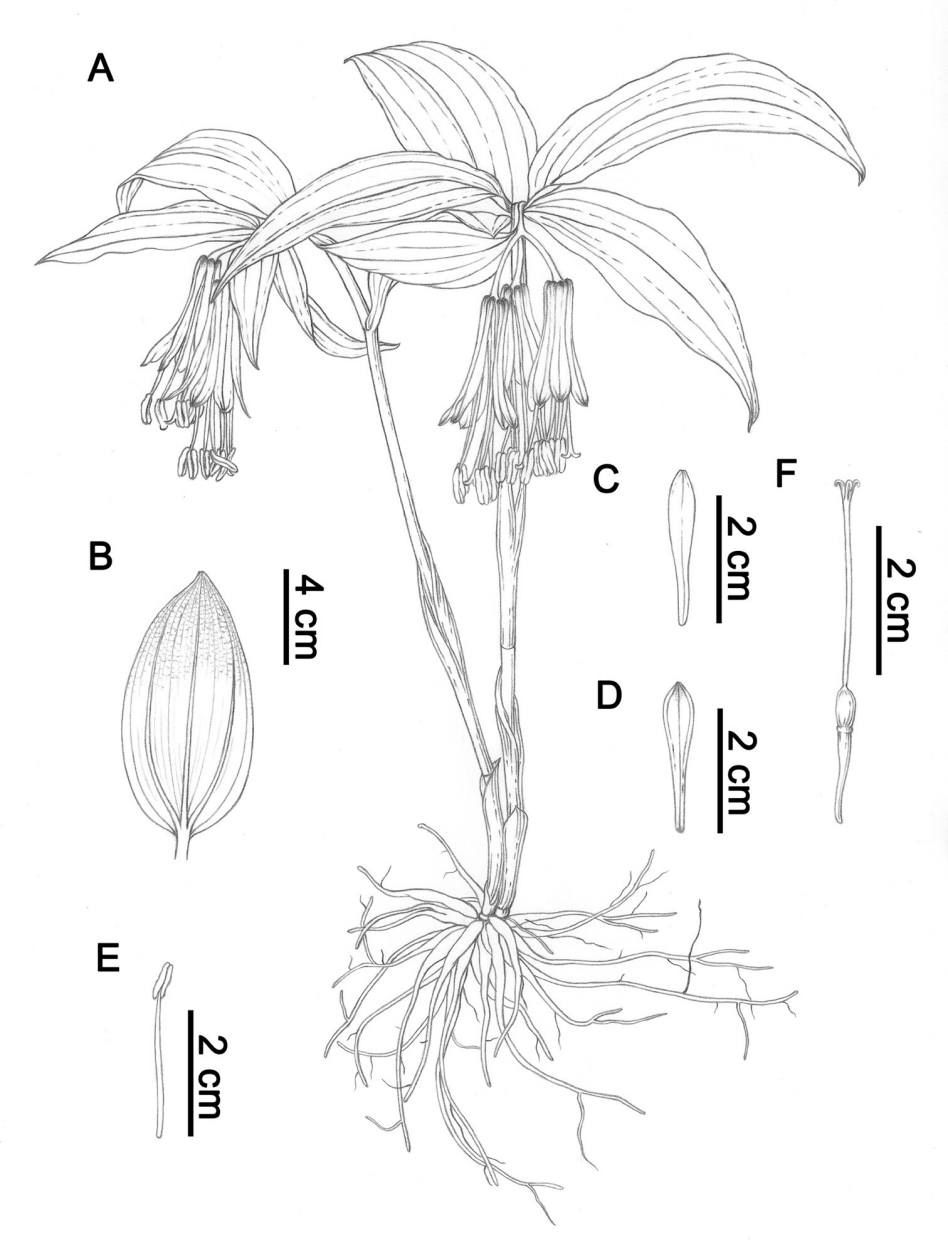
*Disporum nanchuanense* X.X.Zhu & S.R.Yi. **A** Plant **B** Leaf **C** Tepal in frontal view **D** Tepal in back view **E** Stamen **F** Pistil. Illustration by Huixia Dong.

**Figure 2.**
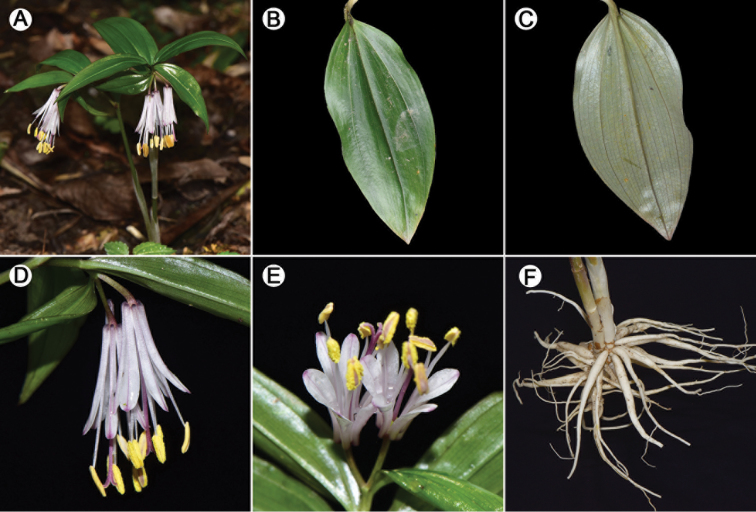
*Disporum nanchuanense* X.X.Zhu & S.R.Yi. **A** Plant **B–C** Leaves **D–E** Inflorescences **F** Roots. Photographed by Xinxin Zhu

**Figure 3.**
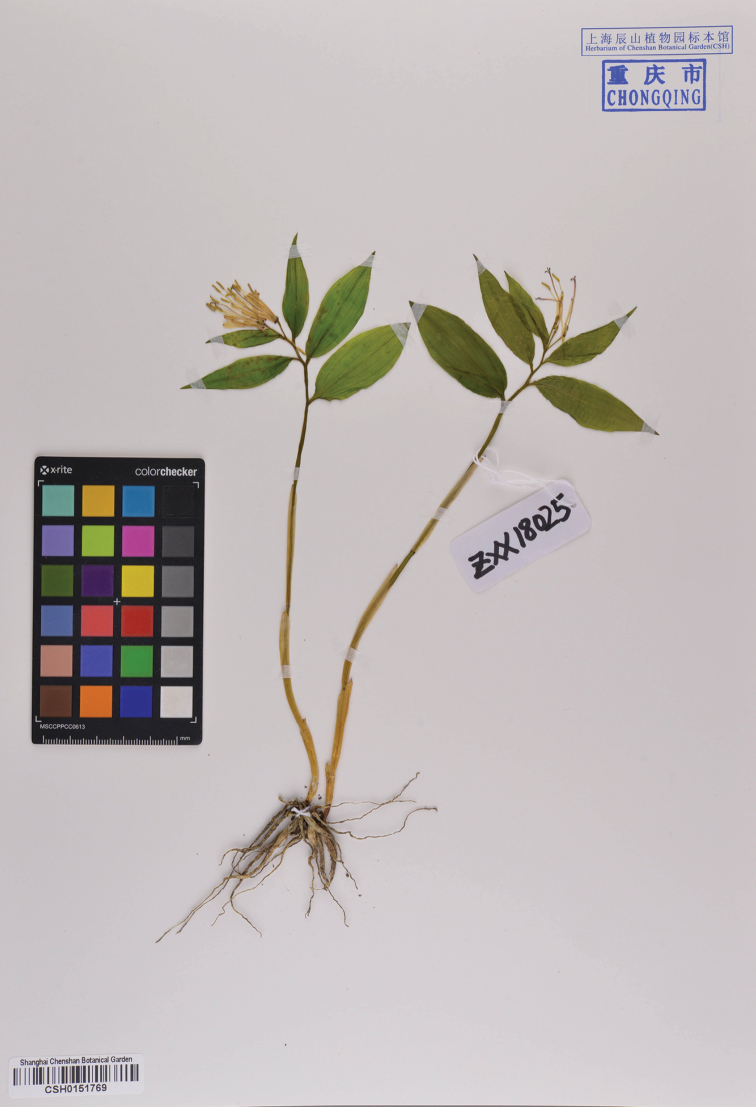
Holotype of *Disporum nanchuanense* X.X.Zhu & S.R.Yi (CSH-0151769!).

**Figure 4.**
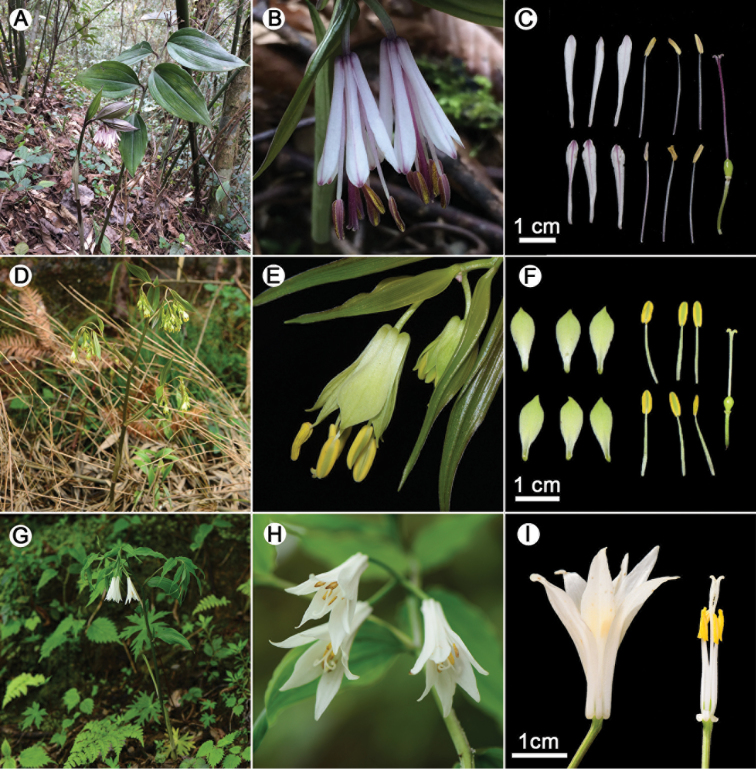
**A-C**: *Disporum nanchuanense* X.X.Zhu & S.R.Yi. **A** Plant **B** Inflorescence **C** Flower dissection **D-F**: *D. longistylum* (Lévl. & Vant.) Hara **D** Plant **E** Inflorescence **F** Flower dissection **G-I**: *D. megalanthum* Wang & Tang **G** Plant **H** Inflorescence **I** Flower dissection. **A-H**: Photographed by Xinxin Zhu; I: Photographed by Renbin Zhu.

Figures 1 , 2 , 3 and 4A-C

#### Type.

CHINA. Chongqing: Nanchuan District, Jinfo Mountain National Nature Reserve, 29°02.67'N, 107°11.32'E, 1386 m a.s.l., 30 March 2018, *X.X.Zhu ZXX18025* (holotype: CSH [CSH 0151769!]; isotypes CSH!, KUN!).

#### Diagnosis.

*Disporum nanchuanense* X.X.Zhu & S.R.Yi is similar to *D. longistylum* (Léveillé & Vaniot) Hara and *D. megalanthum* Wang & Tang, but it differs from *D. longistylum* in its stem which is usually simple, rarely branched, tepals white with purple, apex obtuse, 22–25 × 2.8–4 mm, filaments 20–24 mm long, anthers 5.2–5.6 mm long and ovary ca. 4.6 mm long, style 27–29 mm long; and it differs from *D. megalanthum* in its inflorescences terminal, petiole 3–13 mm long, tepals white with purple, oblanceolate, apex obtuse, stamens distinctly exserted and ovary ca. 4.6 mm long, style 27–29 mm long, distinctly exserted. Detailed morphological comparison is shown in Table 1 and Figure 4.

**Table 1.** Morphological comparisons amongst *Disporum nanchuanense*, *D. longistylum* and *D. megalanthum*.

**Characters**	***D. nanchuanense***	***D. longistylum***	***D. megalanthum***
Stem	usually simple, rarely branched, 20–45 cm	usually branched distally, 30–100 cm	usually slightly branched distally, 30–60 cm
Inflorescence	terminal	terminal	opposite to a leaf or terminal on a short lateral branchlet
Petiole	3–13 mm long	3–10 mm long	2–4 mm long
Tepals	white with purple, oblanceolate, apex obtuse, 22–25 × 2.8–4 mm	green or greenish-yellow, spatulate-oblanceolate to obovate, apex subacute, 10–17 × 2–4(–8) mm	white, obovate-oblanceolate, apex acute, rarely obtuse, 20–38 × 5–8 mm
Stamens	distinctly exserted	distinctly exserted	included
Filaments	20–24 mm long	10–16 mm long	14–22 mm long
Anthers	5.2–5.6 mm long	2.5–4.5 mm long	4–6 mm long
Ovary	ca. 4.6 mm long	2–4 mm long	2–3 mm long
Style	27–29 mm long, distinctly exserted	8–17 mm long, exserted	12–18 mm long, included

#### Description.

Perennial herb with short rhizomes; occasionally with stolons. Roots densely tufted, fleshy. Stem usually simple, rarely branched, 20–45 cm high, proximally with several sheaths. Leaves alternate, concentrated in distal part of stem; petiole 3–13 mm long; blade ovate-lanceolate to oblong, papery to thinly leathery, 6.5–12.5 × 1.1–6 cm, apex shortly acuminate to acute, base broadly cuneate or roundish, glabrous, 3–5-nerved. Inflorescence terminal, 2–7-flowered, non-pedunculate. Pedicels 10–13 mm long. Flowers narrowly campanulate, nodding; tepals 6, oblanceolate, white with purple, glabrous on both surfaces, minutely papillose on the lower margin, 22–25 × 2.8–4 mm, apex obtuse, base gibbous-spurred; spurs ca. 1.5 mm long. Stamens inserted at the base of tepals, distinctly exserted; filaments glabrous, 20–24 mm long, anthers 5.2–5.6 mm long. Ovary oblong, green, glabrous, ca. 4.6 mm long; style glabrous, 27–29 mm long, distinctly exserted, trifid at the apex, branches 3, densely papillose inside.

#### Phenology.

Flowering from March to April. No fruiting specimens have been seen.

#### Etymology.

The specific epithet refers to the type locality, Nanchuan District, Chongqing, China. The Chinese name is given as “南川万寿竹”.

#### Distribution and habitat.

*Disporum nanchuanense* is presently known only from the type locality in Jinfo Mountain National Nature Reserve, Nanchuan District, Chongqing. It grows under conifer-broadleaved forest at 1386–1411 m, together with *Arisaema bockii* Engl. (Araceae), *Cardamine hygrophila* T.Y.Cheo & R.C.Fang (Brassicaceae), *Cephalotaxus fortunei* Hook. (Cephalotaxaceae), *Liriodendron chinense* (Hemsl.) Sargent. (Magnoliaceae), *Pinus massoniana* Lamb. (Pinaceae), *Rhododendron coeloneurum* Diels (Ericaceae), *Sanicula orthacantha* S.Moore (Apiaceae) etc.

#### IUCN Red List category.

*Disporum nanchuanense* is known from only one population, with fewer than fifty individuals seen at this site. Therefore, the new species is assigned a preliminary status of Vulnerable (VU D2), according to IUCN Red List criteria, indicating a population with a very restricted area of occupancy (typically less than 20 km^2^) or number of locations (typically five or fewer).

#### Specimens Examined.

**(Paratypes). CHINA. Chongqing:** Nanchuan District, Jinfo Mountain National Nature Reserve, 28 March 2017, *S.R.Yi ZXX17109* (CSH!, KUN!).

### Key to the 18 species of *Disporum* in China

**Table:** 

1	Tepals dark purple to purplish or yellow	**2**
2	Tepals dark purple to purplish	**3**
3	Tepals long spurred, spurs cylindric, 4–5(–8) mm	***D. calcaratum***
3'	Tepals shortly spurred, spurs gibbous, 1–3 mm	***D. cantoniense***
2'	Tepals yellow	**4**
4	Leaf blade broadly elliptic to oblong-ovate; filaments minutely papillose proximally; Anhui, Chongqing, Hebei, Hubei, Jiangsu, Jiangxi, Liaoning, Shaanxi, Shandong, Sichuan, Zhejiang	***D. uniflorum***
4'	Leaf blade linear-lanceolate to oblong-lanceolate; filaments glabrous; Taiwan	***D. shimadae***
1'	Tepals white to cream, sometimes purple red distally	**5**
5	Tepals minutely puberulent on both surfaces, apex long acuminate	***D. acuminatissimum***
5'	Tepals glabrous on both surfaces, apex obtuse to acuminate	**6**
6	Flowers tubular-campanulate, not widely opening; tepals broadest in the upper part	**7**
7	Tepals smaller, 5–10 mm long	***D. hainanense***
7'	Tepals larger, 10–38 mm long	**8**
8	Leaves rather thick, subleathery, with distinct cross veins	***D. trabeculatum***
8'	Leaves thinner, herbaceous to thinly leathery, without distinct cross veins	**9**
9	Stamens and pistil distinct exserted; tepals larger, 10–25 mm long	**10**
10	Tepals white with purple, oblanceolate, apex obtuse, 22–25 mm long; filaments 20–24 mm long; style 27–29 mm long	***D. nanchuanense***
10'	Tepals green or greenish-yellow, spatulate-oblanceolate to obovate, apex subacute, 10–17 mm long; filaments 10–16 mm long; style 8–17 mm long	***D. longistylum***
9'	Stamens and pistil shorter than tepals	**11**
11.	Tepals larger, 20–38 mm long, white, apex acute, rarely obtuse; Chongqin, Guizhou, Hubei, Shaanxi, Sichuan	***D. megalanthum***
11.	Tepals smaller, 15–22 mm long, white with green tips and violet spots or cream with red spot at tips, apex blunt tip; Taiwan	**12**
12	Plant deciduous, with stolon; inflorescences all truly terminal; tepals white with green tips and violet spots	***D. nantouense***
12'	Plant evergreen, without stolon; inflorescence pseudoterminal; tepals cream with red spot at tips	***D. kawakamii***
6'	Flowers obconic to turbinate, widely opening; tepals broadest in the lower or middle part	**13**
13	Roots densely puberulent; tepals larger, 23–31 mm long	**14**
14	Leaf blade narrowly lanceolate, thinly leathery; tepals white, oblong, broadest in the middle part, lower part not navicular-scaphoid	***D. sinovietnamicum***
14'	Leaf blade ovate to elliptic, papery or herbaceous; tepals greenish-yellow or white, lanceolate, broadest in the lower middle, lower part navicular-scaphoid	***D. xilingense***
13'	Roots glabrous; tepals smaller, 10–18 mm long	**15**
15	Stem often branched distally, 30–80 cm high, with 3–7 leaves below branching; tepals are 2–3 times as long as stamens; filaments as long as anthers	***D. viridescens***
15'	Stem simple or few-branched, 15–35 cm high, with 0–2 normal leaves below branching; tepals are 1.5–2 times as long as stamens; filaments are 2 times as long as anthers	**16**
16	Leaves 4–9 all on the upper 2/3 to whole part of the stems; petioles less than 2 mm long; Shandong, Korean Peninsula, Japan	***D. smilacinum***
16'	Leaves 3–4 all on the upper 1/3 part of the stems; petioles 2–4 mm long; Chongqing, Guizhou, Hunan, Sichuan, Yunnan	**17**
17	Tepals greenish-yellow, (6)8–12 mm long; stamens (5)6–8 mm long; filaments 3–4.5 mm long	***D. bodinieri***
17'	Tepals white, 13–17 mm long; stamens 8–11 mm long; filaments 5–7 mm long	***D. jinfoshanense***

## Discussion

*Disporum nanchuanense* is morphologically similar to *A. longistylum* and *A. megalanthum*. However, the new species differs from the two species in vegetative and reproductive characters, which have been summarised in Table 1, as well as those shown in Figure 4.

Though *Disporum* comprises only 24 species, the genus is still taxonomically problematic and many species are difficult to identify, with either diagnostic characters too variable and/or obscure or having been provided with incomplete descriptions due to a lack of sufficient field investigations (Hara 1984; Hara 1988; Hareesh et al. 2018). In an effort to achieve a better understanding of the taxonomy of *Disporum*, we have been working on the genus since 2016. Plants from 16 *Disporum* species from ca. 100 populations have been well observed and collected in the field during the past two years, especially for species from China. About 3800 specimens from 35 herbaria (AU, BM, BNU, CDBI, CIB, CSH, E, HENU, HHBG, HITBC, HTC, HX, IBK, IBSC, IFP, K, KUN, KYO, LBG, MO, NAS, NEFI, NKU, NWAFU, NY, P, PE, PEM, QTPMB, SCUM, SZ, TAI, TI, US and WUK; abbreviations follow Thiers in Index Herbariorum available at http://sweetgum.nybg.org/science/ih/) have been carefully checked. Here, a new identification key to 18 species of *Disporum* in China is provided based on our study and previous publications (Léveillé 1905; Hara 1988; Liang and Tamura 2000; Li et al. 2007; Hu et al. 2016; Zhu et al. 2016), in order to better evaluate of the position of the new species in *Disporum*.

## Supplementary Material

XML Treatment for Disporum nanchuanense
